# The metabolic footprint of the airway bacterial community in cystic fibrosis

**DOI:** 10.1186/s40168-017-0289-z

**Published:** 2017-06-30

**Authors:** Vaishnavi Narayanamurthy, John M. Sweetnam, Darcy R. Denner, Lena W. Chen, Edward T. Naureckas, Bharathi Laxman, Steven R. White

**Affiliations:** 1 Department of Medicine, Section of Pulmonary and Critical Care Medicine, The University of Chicago, Chicago, IL 60637 USA; 2 Present address: Medical College of Georgia, Augusta University, Augusta, GA 30912 USA

**Keywords:** Cystic fibrosis (CF), Microbiome, Bacteria, Oxidative activity, Community-level physiological profiling (CLPP), 16S sequencing, Biolog Gen III Microplate, Pseudomonas

## Abstract

**Background:**

Progressive, chronic bacterial infection of the airways is a leading cause of death in cystic fibrosis (CF). Culture-independent methods based on sequencing of the bacterial 16S rRNA gene describe a distinct microbial community that decreases in richness and diversity with disease progression. Understanding the functional characteristics of the microbial community may aid in identifying potential therapies and may assist in management, but current methods are cumbersome. Here, we demonstrate the use of an oxidative metabolic assay as a complement to sequencing methods to describe the microbiome in the airways of patients with CF.

**Methods:**

Expectorated sputum was collected from 16 CF subjects and 8 control subjects. The Biolog Gen III Microplate was used in a community-level physiological profiling (CLPP)-based assay to examine oxidative metabolic activity. 16S rRNA V4 amplicon sequencing was used to characterize the taxonomy and diversity of the samples. Correlations were then identified among the oxidative activity and taxonomy data. In an additional paired analysis, sputum from seven CF subjects were collected at two separate clinic visits and compared for oxidative activity, taxonomy, and diversity.

**Results:**

Significant differences in oxidative metabolic activity, microbial taxonomy, and diversity were found between the CF and control sputum samples. Oxidative activity correlated positively with total genera but not with other measures of diversity or taxonomy, demonstrating that the metabolic assay complements the structural aspects of the microbiome. As expected, *Pseudomonas* was significantly enriched in CF samples, while *Streptococcus* and *Prevotella* were similarly abundant in both CF and control samples. Paired analysis of CF samples at separate clinic visits revealed comparable oxidative activity that correlated with similar stability in taxonomy and diversity.

**Conclusions:**

The CLPP assay used in this study complements existing sequencing methods to delineate the oxidative metabolic footprint of the CF airway bacterial community. This method may be useful to study the CF microbial community over time and with changes in disease state.

**Electronic supplementary material:**

The online version of this article (doi:10.1186/s40168-017-0289-z) contains supplementary material, which is available to authorized users.

## Background

Chronic respiratory tract infection combined with airway inflammation leads inexorably to respiratory failure and death in patients with cystic fibrosis (CF) [1]. Cross-sectional and longitudinal studies using culture-independent analyses employing the sequencing of the bacterial 16S rRNA gene have identified a complex microbial community [2–4] that decreases in both diversity and richness with disease progression [3, 5–11]. Much work has focused both upon individual species and the taxonomy of the community found in periods of clinical stability, during/after clinical exacerbations, and in correlation with worsening lung function and clinical status [3, 8, 10, 12, 13].

Assays for community-level physiological profiling (CLPP) of cultivable microbial populations [14–17] have been used to examine the function of microbial communities in wetlands, soil, and agricultural applications as well as in microbial assays for species identification. CLPP is a simple technique to characterize cultivable, functionally mixed microbial communities based on carbon source utilization assays. While the assay has been used in some studies to identify isolated pathogens from human clinical samples [18–20], to date, only one case report has used CLPP technology to examine the function of a microbiome in human subjects with CF [21].

In this study, we extend the use of CLPP to study the oxidative metabolic activity of the airway microbiome in patients with CF. We demonstrate the utility of this method to compare functional oxidative metabolism to the taxonomy and diversity of the airway microbiome in CF subjects.

## Results

### Demographics and clinical characteristics

Sixteen adult subjects between the ages of 21 and 62 years were recruited for the CF cohort. All CF subjects except two were receiving systemic or inhaled antibiotics at the time of recruitment. The control cohort consisted of 8 healthy subjects between the ages of 20 and 59 without known lung disease. Subjects’ characteristics are summarized in Table 1. The CF subjects’ genotype and therapy are provided in detail in Additional file 1. As expected, CF subjects had a lower FVC (*p* = 0.003) and FEV1 (*p* = 0.001) compared to control subjects. CF subjects also had higher absolute blood neutrophil counts (*p* = 0.005) and percent of neutrophils in the white blood cell count (*p* = 0.007) compared to control subjects. Absolute blood eosinophil counts and serum IgE concentrations did not differ significantly between the CF and control subjects.

**Table 1:** Demographics and clinical parameters of study subjects

Parameter(s)	CF (*n* = 16)	Control (*n* = 8)	*P* value
Age, years	31.0 (25.5, 36.0)	33.5 (26.5, 46.5)	NS^*^
Sex (male:female)	10:6	0:8	0.006^†^
Race (EA:AA:other)	15:1:0	1:7:0	0.001^†^
Antibiotic use (yes:no)	14:2	0:8	NA^§^
Serum IgE, U/mL	77.5 (29.5, 119.5)	72.5 (23.0, 201.0)	NS
Absolute blood eosinophils	0.16 (0.11, 0.26)	0.11 (0.06, 0.18)	NS
FVC, % predicted	84.0 (59.5, 98.5)	108.0 (99.0, 113.5)	0.003
FEV1, % predicted	72.5 (55.5, 87.5)	106.0 (95.5, 112.0)	0.001
Neutrophils, % of total WBC	71.5 (63.0, 76.0)	58.5 (49.0, 62.5)	0.007
Absolute blood neutrophils	6.29 (3.77, 8.73)	2.77 (1.89, 4.22)	0.005
CF genotype (for full details, see Additional file 1)			
Processing defect (F508del homo/heterozygous)	11	ND^⌘^	NA
Gating mutation (G551D)	3	ND	NA
Non-sense mutation (R1158X, G542X)	2	ND	NA

Proportions are shown as median (IQR). *P* values were generated from Wilcoxon-Mann-Whitney rank sum test unless otherwise stated

^*^Not significant

^†^ *P* value by Fisher’s exact test

^§^Not applicable

^⌘^Not determined

The demographic details of the seven CF subjects used in the additional paired analysis are summarized in Additional file 2. The clinical parameters (FVC, FEV1, blood eosinophil counts, serum IgE concentrations, and blood neutrophil counts) did not differ between visits.

### CF samples have lower oxidative activity

Oxidative metabolic activity of the airway microbial communities from CF and control subjects was assessed using the Biolog Gen III Microplate. After considering how best to normalize data, raw optical density (OD) values were corrected with the starting bacterial density measured at 600 nm and were expressed as corrected average well color density (AWCD). Additional file 3 shows a positive correlation between the starting bacterial density values and the raw optical density of the positive control wells on the plates.

**Fig. 1:**
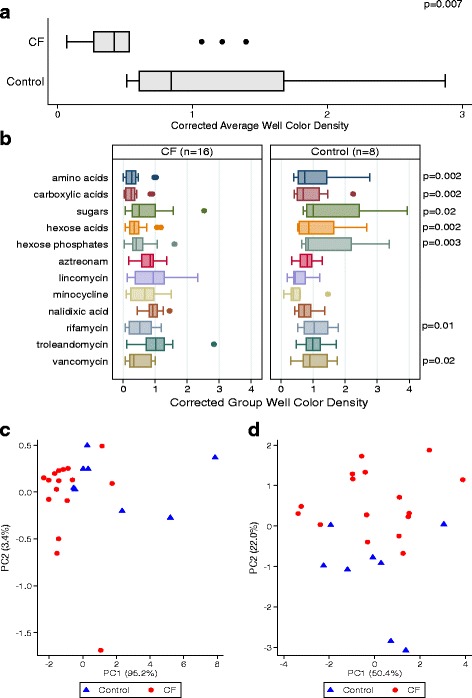
Oxidative metabolic activity of sputum samples from 16 CF and 8 control subjects. **a** Average well color density (AWCD) of metabolic activity corrected for starting bacterial density. *P* value was obtained using the Wilcoxon-Mann-Whitney rank sum test. **b** Corrected group well color density values of individual carbon source and antibiotic assays in CF and control samples. *P* values were generated using the Wilcoxon-Mann-Whitney rank sum test. Only significant values (<0.05) are displayed. **c** Principal component analysis of corrected AWCD in the presence of carbon sources in both sets of subjects. **d** Principal component analysis of corrected AWCD in the presence of antibiotics in both sets of subjects. Only modest separation between CF and control samples is noted in both PCA plots

The bacterial communities from the CF samples demonstrated significantly lower AWCD (*p* = 0.007) as well as significantly lower group well color densities in the presence of different carbon sources (amino acids, carboxylic acids, sugars, hexose acids, and hexose phosphates) (Fig. 1a, b). However, oxidative activity in the presence of assay antibiotics was varied. The optical density of the CF samples in the presence of troleandomycin (a macrolide similar to azithromycin commonly used in patients with severe CF) and aztreonam (frequently used to treat *Pseudomonas* infections) were not different from the control samples (Fig. 1b). Conversely, both rifamycin and vancomycin decreased the optical density in the CF samples when compared to control. A principal component analysis (PCA) done on the oxidative activity data demonstrated modest separation between control and CF samples for both carbon sources and antibiotics (Fig. 1c, d).

The paired analysis conducted on the CF samples comparing the microbial communities at two separate clinic visits demonstrated no significant changes in oxidative activity (Additional file 4).

### Sequencing and composition

**Fig. 2:**
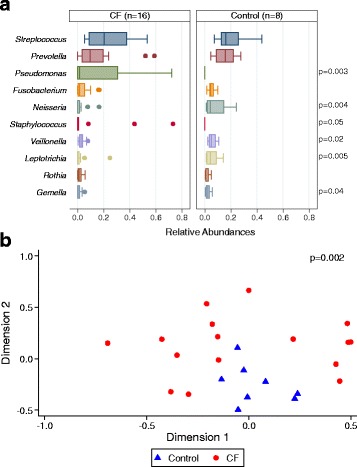
Relative abundance of 10 most abundant genera in 16 CF and 8 control samples. **a** Relative abundances of genera in samples. *P* values were generated using the Wilcoxon-Mann-Whitney rank sum test. **b** Principal coordinate analysis by non-metric multidimensional scaling generated from Bray-Curtis dissimilarity matrices at the genus level from CF and control samples. *P* value was obtained using HOMOVA

DNA sequencing after quality filtering yielded 1,377,485 sequences, which were classified into operational taxonomic units (OTUs). The sequence count distribution for the experimental samples is 57379 ± 20294 (SD); the minimum was 34429 and maximum was 137358. Subsampling was done with the size set to that of the sample with the lowest sequence count, 34429. The OTUs were classified into 265 total genera; however, the bottom 189 genera each represented less than 0.01% of the total. The top 18 genera accounted for 90% of all sequences, and *Streptococcus*, *Prevotella*, and *Pseudomonas* accounted for over half (52%) of the sequences. The relative abundances of *Pseudomonas* and *Staphylococcus* were significantly enriched in CF samples; *Neisseria*, *Veillonella*, and *Leptotrichia* were enriched in control samples, while both *Streptococcus* and *Prevotella* were similar in CF and control samples (Fig. 2). The samples from each subject separated into clusters in a Bray-Curtis-based non-metric multidimensional scaling (NMDS) ordination plot (Fig. 2). Samples from control subjects occupied a similar space on the ordination plot, whereas the samples from CF subjects clustered less well, suggesting differences in dominant genera both within this group and compared to control subjects. Homogeneity of molecular variance (HOMOVA) was used to determine if subjects differed significantly from each other with respect to overall variability in community structure. Greater variation in community structure was noted in the CF subjects (*p* = 0.002) compared to controls.

Analysis of paired CF samples revealed no significant differences in relative abundance of the most abundant genera. Examination of an NMDS ordination plot suggested that the paired samples did not separate into clusters (Additional file 5). The Chao1 index, inverse Simpson index, Shannon diversity index, and the numbers of genera did not differ between the two sets of samples (Additional file 6). These data suggested that the CF microbiome remained stable with reference to taxonomy and diversity during periods of clinical stability.

### CF samples have lower diversity and richness

**Fig. 3:**
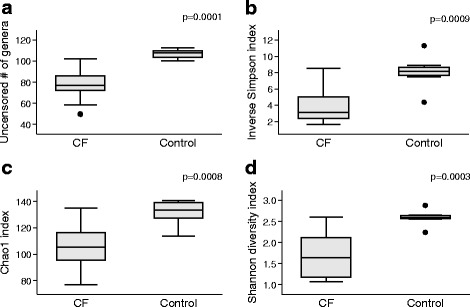
Analysis of diversity and richness of microbiome in 16 CF and 8 control samples. Panels show uncensored number of genera, inverse Simpson index, Chao1 index, and Shannon diversity Index. *P* values were generated using Wilcoxon-Mann-Whitney rank sum tests

As expected, the CF samples were found to have significantly lower alpha diversity and richness when compared to controls as measured by the uncensored number of genera (richness), inverse Simpson (diversity), Chao1 (richness), and Shannon (diversity) indices (Fig. 3). Analysis of the paired microbial communities revealed no significant differences in diversity and richness (Additional file 6).

### Lack of correlation of oxidative activity to measures of taxonomy and diversity

**Fig. 4:**
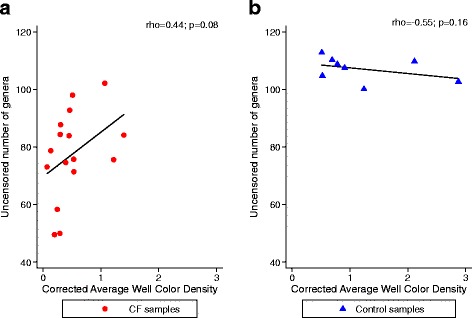
Correlations between oxidative activity (AWCD) and uncensored number of genera. *P* and rho values were obtained using the Spearman’s correlation test. The CF and control samples are shown separately

We examined whether oxidative activity was related to the relative abundance of select genera, and to measures of richness and diversity, in the samples. Corrected AWCD had but a loose correlation when compared to uncensored number of genera in CF samples, and no correlation in control samples (Fig. 4). However, there was no significant correlation between oxidative activity and the Chao1, Shannon, or inverse Simpson indices, nor to the relative abundance of *Pseudomonas*, *Veillonella*, *Prevotella*, and *Streptococcus*. These data suggested that the measures of the cultivable and total communities examined different, non-correlated aspects of the community.

### Oxidative activity of CF microbiome compared to Pseudomonas aeruginosa (PA) in culture

**Fig. 5:**
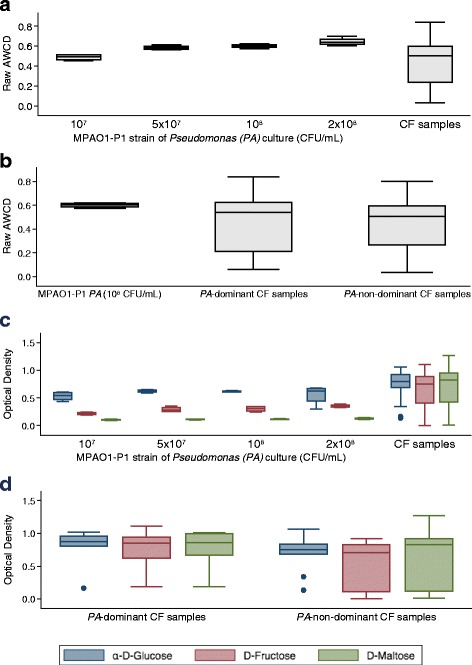
Comparison of oxidative activity of CF microbiome to pure *Pseudomonas aeruginosa* (*PA*). **a** Raw average well color density (*AWCD*) from CF samples (*n* = 16) compared to the raw AWCD from four different concentrations of MPAO1-P1 strain of *PA* (*n* = 4 different experiments). **b** CF samples were stratified into *PA*-dominant and *PA*-non-dominant and were compared against the MPAO1-P1 strain at 10^8^ CFU/mL. No significant differences were noted. **c** Optical density for tetrazolium reduction in CLPP assay wells containing α-D-glucose, D-fructose, or D-maltose from the four different concentrations of MPAO1-P1 strain above (*n* = 4 experiments) and for the 16 CF samples. **d** Optical density for tetrazolium reduction in CLPP assay wells containing α-D-glucose, D-fructose or D-maltose for *PA*-dominant and *PA*-non-dominant CF samples

We then asked how the oxidative activity of the CF microbiome compared to that of one common species in the CF airway, *Pseudomonas aeruginosa* (*PA*), grown in pure culture (MPAO1-P1 strain). As the concentration of the PA-MPAO1-P1 strain increased, the raw AWCD also increased (Fig. 5a). CF samples then were stratified into *PA*-dominant and *PA*-non-dominant based on positive or negative growth of *PA* cultures in clinical microbiology reports from samples at the time of clinic visits. These *PA*-dominant CF samples also indicated a high level of *Pseudomonas* from sequencing data (relative abundance > 20% of total sequences). No significant differences were found in the oxidative activity between the pure *PA* culture, *PA*-dominant, and *PA*-non-dominant CF samples (Fig. 5b).

As *Pseudomonas* and other gram-negative organisms may have very different fermentative capacity than that seen with oral microbes (e.g., *Prevotella* and *Veillonella*), we examined the use of substrates such as α-D-glucose, D-fructose, or D-maltose in the CLPP assay. The MPAO1-P1 strain utilized α-D-glucose and D-fructose but not D-maltose in these experiments, whereas the CF microbial communities utilized each of these substrates (Fig. 5c). These data suggested that the other members of the microbial community present in the CF samples had a more substantial contribution to overall metabolism in the presence of these sugars, compared to MPAO1-P1 alone. We also examined the data from the CF patients as segregated by *PA*-dominant and non-dominant samples. For each of the three sugars, the CF community responses were similar regardless of the dominance of *PA*, though the non-dominant samples demonstrated more variance (Fig. 5d).

## Discussion

In this study, we demonstrated the use of a CLPP-based metabolic assay as a complement to existing 16S sequencing methods to describe the microbiome in the airways of CF subjects. Using the Biolog Gen III Microplate, we compared the microbial communities in the airways of CF and control subjects at a functional level. We then drew correlations to taxonomy data derived from 16S sequencing. We found that the oxidative activity correlated positively to the total number of genera but not to other measures of taxonomy or diversity, suggesting that the measures of the cultivable and total communities examined complimentary, non-correlated aspects of the community. We also conducted a paired analysis of the microbial communities in the airways of CF subjects at two separate clinic visits at times of clinical stability. The oxidative activity, diversity, and richness remained similar at both visits. This further underscores the utility of the metabolic assay. To the best of our knowledge, this is the first study to use the CLPP assay to describe functional metabolism of a cultivable bacterial community in human subjects with CF.

We found a significant difference in the oxidative metabolic activity between CF and control sputum samples. The oxidative activity of the CF samples was significantly reduced in the presence of various select carbon sources, while the activity was varied in the presence of select antibiotics. Since all but three of the CF subjects were receiving systemic antibiotics, and several were receiving inhaled antibiotics, at the time of sample collection, this reduced oxidative activity may be due to the suppressive effect of these agents. Customizing the assay to include antibiotics commonly used in treatment may have practical application in a clinical setting. As one example, an individual patient’s antibiotic sensitivities, based on oxidative metabolism, could be tested rapidly using the CLPP assay.

All samples from the CF subjects were obtained during regularly scheduled clinic visits, when the subjects were judged to be clinically stable. Although some CF subjects in the paired analysis were hospitalized due to CF exacerbations between their clinic visits, their microbiomes appeared to recover with respect to both function and composition. An interesting question to be answered in future studies is whether the CLPP oxidative assay changes significantly during periods of exacerbation, and whether these changes reflect underlying changes in taxonomy and relative abundance of pathogens. Such an assay, performed quickly at the time at or near the onset of a CF exacerbation, may help guide therapy.

One consideration relates to the selection of carbon sources, environmental stresses, and antibiotics selected for inclusion in the Biolog Gen III Microplates. These were originally selected based on their usefulness in varying ecological studies (e.g., studies of soil microbiota) and may not be optimized for the study of oxidative metabolism in human-associated microbiomes. We analyzed logical subgroups of carbon sources, such as carboxylic acids, hexose phosphates, and sugars. These subgroups performed similarly to analyses done using the average well color density (AWCD) values corrected for starting bacterial density in demonstrating differences between the CF and control microbial communities. Substitution of different carbon sources, antibiotics, or environmental stressors may yield other results, and such substitution may be useful in future studies that seek a more fine-grained understanding of bacterial metabolism in human microbiomes in disease states.

The CLPP assay used in this study differs substantially from the complex microenvironment of sputum and the sol layer (the aqueous portion of the airway secretions that line the respiratory tract) of the lower airways. The assay here tested the ability of microbes to oxidize tetrazolium in the presence of single substrates in simple medium. In contrast, in the living state many substrates are available, many breakdown products that may be of use (or that may hinder growth) are present, and oxygen tension and redox potential may differ from site to site within an airway or within the sputum [22–25]. Cowley et al. [22] for example, demonstrated that the sputum in pediatric patients with CF is dynamic, frequently anoxic, and is frequently reduced with low pH and high redox potential. More complex assays to measure oxidation in the presence of these conditions, and with multiple substrates, will help understand better the complexities of the microbial community in CF airways.

Our study has several limitations. All CF subjects were recruited from a single clinic at a single major metropolitan medical center. All CF genotypes have not been accounted for, and differences in location, household exposures, and lifestyle may lead to significant variation in the microbiome. In addition, subjects were on different antibiotics at the time of sample collection, and as noted above, antibiotic usage may have varying effects on oxidative activity as well as taxonomy and diversity.

There are some limitations with the processing of sputum. Some components of sputum, or the dithiothreitol agent used to dissolve sputum mucins, may interfere with the CLPP assay. Since sputum was collected from both control and CF subjects, it is possible that any interference from sputum components, if present, is similar. The CF sputum, however, may be different and may interfere with the assay differently. In addition, the CF bacterial communities collected from sputum may have adapted over time to the local airway environment and thus may oxidize tetrazolium differently than they would oxidize similar substrates in vivo. In support of this, Cowley et al. have demonstrated that sputum samples collected from pediatric patients with CF are significantly anoxic and extremely reduced due to hydrogen sulfide formation [22]. The local environment thus may be very different than the aerobic environment in a simple medium as is found in the CLPP assay. These differences will need to be investigated in future studies to examine the relation of metabolic activity of the microbial communities in CF under these conditions.

Another concern is the ability to identify *Burkholderia*, an important CF pathogen linked to a more rapid decline in lung function and worsening morbidity and mortality [26]. One previous study has demonstrated that only the PacBio RSII platform, and not the MiSeq platform, was able to identify correctly *Burkholderia* [27]. The absence of this pathogen in our 16S sequencing data may thus be an artifact while the organism, if present, may be contributing to the total metabolic activity of the CLPP assay.

The examination of cultivable communities, as opposed to culture-independent methods, limits analysis in microbial ecology to that which can be cultivated. Culture-independent methods such as 16S sequencing make clear the true extent of community representation and diversity [28] but lack the ability to inform the functional capabilities of the community as a whole. The use of CLPP technology provides one measure of community function, and thus may be useful in further understanding the metabolic footprint of the community and how that may relate to studies of airway inflammation and host-defense responses. Using more intensive culture methods, Sibley et al. [29] were able to identify a larger number of genera than were identified in select media traditionally used in microbiological labs for cultivation of CF bacteria. Further, techniques such as CLPP using the standard Gen III Microplates will be restricted to aerobic or aerotolerant metabolism, though plates that are capable of targeting anaerobic organisms and mechanism are available. To develop the CLPP assay and to examine the potential correlations between taxonomy and bacterial metabolic activity, it will be important to understand the presence of and specific activity of the bacteria that contribute to the oxidation of CLPP plate substrates. Demonstration of these bacteria, and their relation to that demonstrated in the CF airway by 16S sequencing, will help elucidate the roles of the cultivable bacteria.

## Conclusions

In summary, the metabolic assay demonstrated in this study can be used to delineate the oxidative metabolic footprint of the cultivable CF airway bacterial community. Understanding the functional aspects of the airway microbiome might lead to novel therapeutic solutions that will improve the management of CF.

## Methods

### Subjects

Sixteen adult subjects with CF and 8 control subjects were recruited. Approval was obtained from the Institutional Review Board at the University of Chicago. All subjects provided written informed consent to the use of biological samples and clinical data.

### Sample collection

Expectorated sputum was collected from CF subjects at the time of a regularly scheduled clinic visit. Subjects with communicable diseases including HIV, tuberculosis, and hepatitis were excluded from study. Control subjects were healthy at the time of sample collection with normal pulmonary function as determined by clinical examination, a respiratory diseases questionnaire and spirometry. No control subjects had received antibiotics within 4 weeks of study. Sputum was obtained from control subjects after induction by hypertonic saline nebulization.

Sputum samples were collected from seven CF subjects on two separate clinic visits. While the sample collected during visit one was used for the primary analysis in this study, samples from both visits were used for an additional paired analysis comparing the state of the microbiome between both clinic visits.

### Sample processing

Sputum samples were divided for clinical and research use immediately after collection. Research samples were mixed with a volume of 10% dithiothreitol equal to 1.5× the mass of the sputum and then were heated for 30 min at 37 °C. Samples were then split into two and were centrifuged to remove the bacterial pellets from supernatants. These pellets were then used for CLPP assay and for 16S rRNA sequencing.

### CLPP assay

Biolog Gen III Microplates (Hayward, CA) were used for the CLPP phenotype assay. Vendor directions were followed. Each well of the Biolog Gen III microplate contains either an individual carbon source (e.g., maltose) or an agent used to test chemical sensitivity (e.g., aztreonam). The complete list of substrates can be found in Additional file 7, “Biolog plate contents”. Substrate consumption by the microbial community generates oxidants that then oxidize tetrazolium dye present in the well so as to produce a violet-blue color. Sample pellets were suspended in 1 mL inoculating fluid A (IFA). Absorbance was measured at 600 nm to determine the starting bacterial density. Each sample was then diluted into 10 mL of IFA before adding 100 μL of the solution to each well plate. Plates were incubated at a constant temperature of 33 °C in static conditions and in the presence of no added CO_2_ per protocol provided by Biolog. After an incubation period of 18 h, absorbance was measured at 499 nm using a microplate spectrophotometer.

As the Biolog assay does not have a true control well, 3 plates were used in which 10 mL of plain IFA with no bacteria were used to load wells as before, and then were incubated at 33 °C for 18 h. Absorbance was measured at 499 nm, and the AWCD was calculated. These three blank plates were very uniform with no low background and each of the three plates was in substantial agreement with very low coefficient of variance. Data can be found in Additional file 7, “IFA only (blanks)” sheet. The average AWCD value from these three plates served as the negative control and was subtracted from plates used for CF and control samples. The background subtracted optical density values were then corrected for the starting bacterial density in each plate. Wells containing the chemical sensitivity assays were reported as the ratio to the optical density value from the positive control well. Corrected AWCD for the entire plate was calculated to assess the overall oxidative activity. Group well color density values for each chemical group (i.e., antibiotics, sugars, etc.) were also calculated.

### DNA extraction

Sample pellets were resuspended in 500 μL sterile water and were stored at −80 °C. DNA extractions were done using BiOstic Bacteremia DNA isolation kits (MO BIO Laboratories, Inc., Carlsbad, CA) according to vendor directions. Mechanical disruption of bacterial cells was done using MicroBead tubes and heat. Any antibiotics were neutralized with charcoal and were removed after which DNA was concentrated.

### 16S rRNA amplicon library preparation and sequencing

Processing of the extracted DNA was performed at the National Laboratory for High-Throughput Genome Analysis Core at Argonne National Laboratory (Argonne, IL). All samples first underwent screening for the presence of the 16S rRNA gene within extracted total DNA using PCR and primers specific for the 515-806bpV4 region of the 16S rRNA encoding gene (338 F: 5′-GTGCCAGCMGCCGCGGTAA-3′; 806R: 5′- GGACTACHVGGGTWTCTAAT-3′). Illumina 3′ adapter sequences and a 12 bp barcode were added at that time. Sequencing was done using an Illumina MiSeq DNA sequencer.

### Sequence analysis

Initial sequence processing was done using the MOTHUR suite using the Illumina MiSeq SOP [30, 31] to remove ambiguous bases, too-long sequences, redundancies, and chimeric sequences (the MOTHUR script used can be found in Additional file 8). Sequences were aligned using the SILVA reference database, v119 [32], and were classified into operational taxonomic units (OTU) using a cut-off of ≥97% sequence identity. These then were grouped into appropriate phyla and genera. The number of sequences, observed genera and Good’s coverage per sample were calculated. Community diversity within subjects (alpha diversity) was examined using the uncensored number of genera, inverse Simpson index, the Chao1 index for richness, and the Shannon diversity index. Distances were calculated using the Bray-Curtis measurement. Ordination plots were constructed from dissimilarity matrices using non-metric multidimensional scaling (NMDS). Homogeneity of molecular variance (HOMOVA), a nonparametric analog to Bartlett’s test for homogeneity of variance, was used to determine if subjects differed significantly from each other with respect to overall variability in community structure [33]. Analysis of molecular variance (AMOVA), a non-parametric equivalent to ANOVA, was used to test whether the variation between two samples was different from considering the samples as a single population [34]. Differences in taxonomic distribution of sequences by genus between groups were examined using Metastats with *q* value corrections for false discovery [35].

### Analysis of paired samples

For the additional paired analysis on the samples collected from two separate clinic visits, a separate analysis was done using MOTHUR. Calculations were performed using the same methods as described above.

### Comparison of CF microbiome to pure Pseudomonas (PA) in culture

Biolog Gen III Microplates were used to compare the oxidative activity of the CF samples to that of a culture of a pure virulent *PA* MPAO1-P1 strain [36] (gift of Olga Zaborina, PhD and John Alverdy, MD, PhD, the University of Chicago, and originally obtained from the transposon mutant library at the University of Washington). Sequencing for this strain is available in SRA as accession SRA049017. Four different concentrations of pure *PA* cultures were suspended in IFA and were added to plates. The plates were then incubated and absorbance measured using the same protocol followed for sputum samples. Choosing a concentration where optical densities did not reach saturation, the raw AWCD values for pure *PA* were compared to the raw AWCD values obtained from the sixteen CF samples.

### Data analysis

Clinical data are expressed as median (interquartile range). Initial review of the data from the CLPP assays suggested the lack of a normal distribution. Hence, Wilcoxon-Mann-Whitney non-parametric tests were used with appropriate corrections for multiple comparisons, and Wilcoxon matched-pairs signed-ranks tests were used for paired analyses. Correlations between clinical parameters and either diversity or taxonomy were made using non-parametric Spearman correlations. Statistical analyses were conducted in MOTHUR, STATA (version 14.1, StataCorp, College Station, TX) or R (version 3.0.2, http://www.r-project.org) as required.

## Additional files

Additional file 1: Genotype and therapy of 16 CF subjects. * CF genotype data were previously collected on each subject and identified in medical record review. † Clinical microbiology was generated from a duplicate sputum sample collected at the same time and was analyzed by the clinical microbiology laboratory at the University of Chicago Medicine. (XLSX 49 kb)

Additional file 2: Demographic data of seven CF subjects used in paired analysis. CF genotype data were previously collected on each subject and were identified in medical record review. * CF genotype data were previously collected on each subject and identified in medical record review. (XLSX 34 kb)

Additional file 3: Positive correlation between starting bacterial density and raw optical density of positive control. A positive correlation was noted between the starting bacterial density values obtained at 600 nm and the raw optical density values of the positive control wells. *P* and rho values were obtained using the Spearman’s correlation test. (PDF 18 kb)

Additional file 4: Oxidative activity of sputum samples from seven CF subjects at two clinic visits (paired analysis). Group well color density of metabolic activity corrected for starting bacterial density. *P* values were generated using Wilcoxon matched-pairs signed-rank tests. No significant differences were noted between clinic visits. (PDF 21 kb)

Additional file 5: NMDS ordination plot for seven CF subjects at two clinic visits (paired analysis). Principal coordinate analysis by non-metric multidimensional scaling generated from Bray-Curtis dissimilarity matrices at the genus level from seven CF subjects at two clinic visits. *P* value was obtained using HOMOVA. No separation was noted between the paired samples. (PDF 139 kb)

Additional file 6: Diversity and richness of CF microbiome in subjects at two clinic visits (paired analysis). Panels show uncensored number of genera, inverse Simpson index, Chao1 index, Shannon diversity Index. *P* values were generated using Wilcoxon matched-pairs signed-rank tests. No significant differences were noted between clinic visits. (PDF 17 kb)

Additional file 7: Organization and data from CLPP assays. Complete list of substrates on the CLPP assay and raw data obtained from the CLPP assays in a tabbed Excel (.xlsx) file. The first tab labeled “Notes” describes the contents of the each tab in the worksheet. (XLSX 863 kb)

Additional file 8: MOTHUR script. Provides the MOTHUR script used for initial sequence processing in a Text (.txt) file. (TXT 9 kb)

## Abbreviations

AMOVA: Analysis of molecular variance
ANOVA: Analysis of variance
AWCD: Average well color density
CF: Cystic fibrosis
CFU: Colony-forming unit
CLPP: Community-level physiological profiling
FEV1: Forced expiratory volume in 1 s
FVC: Forced vital capacity
HOMOVA: Homogeneity of molecular variance
IFA: Inoculating fluid A
IgE: Immunoglobulin E
IQR: Interquartile range
MRSA: Methicillin-resistant *Staphylococcus aureus*
MSSA: Methicillin-sensitive *Staphylococcus aureus*
NMDS: Non-metric multidimensional scaling
OD: Optical density
OTU: Operational taxonomic unit
PA: *Pseudomonas aeruginosa*
PCA: Principal component analysis
SA: *Streptococcus agalactiae*
WBC: White blood cell
