# Supplementary figures and images for: The metabolic footprint of the airway bacterial community in cystic fibrosis
Source: Microbiome. 2017 Jun 30;5:67. doi: 10.1186/s40168-017-0289-z (PMC5493850; doi:10.1186/s40168-017-0289-z)

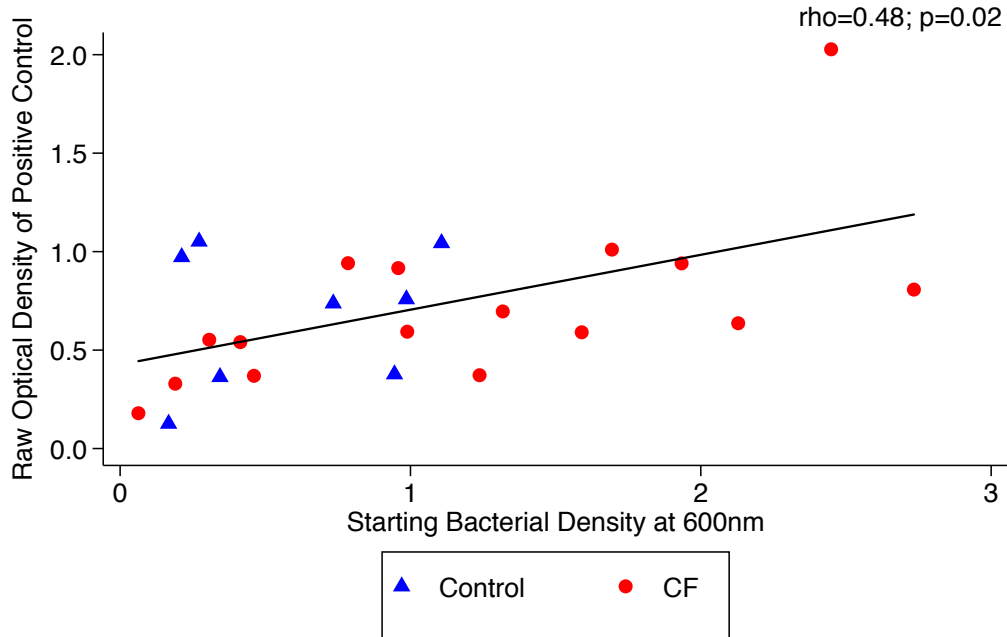

Supplement: Supplementary file 3 — Positive correlation between starting bacterial density and raw optical density of positive control. A positive correlation was noted between the starting bacterial density values obtained at 600 nm and the raw optical density values of the positive control wells. P and rho values were obtained using the Spearman’s correlation test. (PDF 18 kb) [file 40168_2017_289_MOESM3_ESM.pdf]

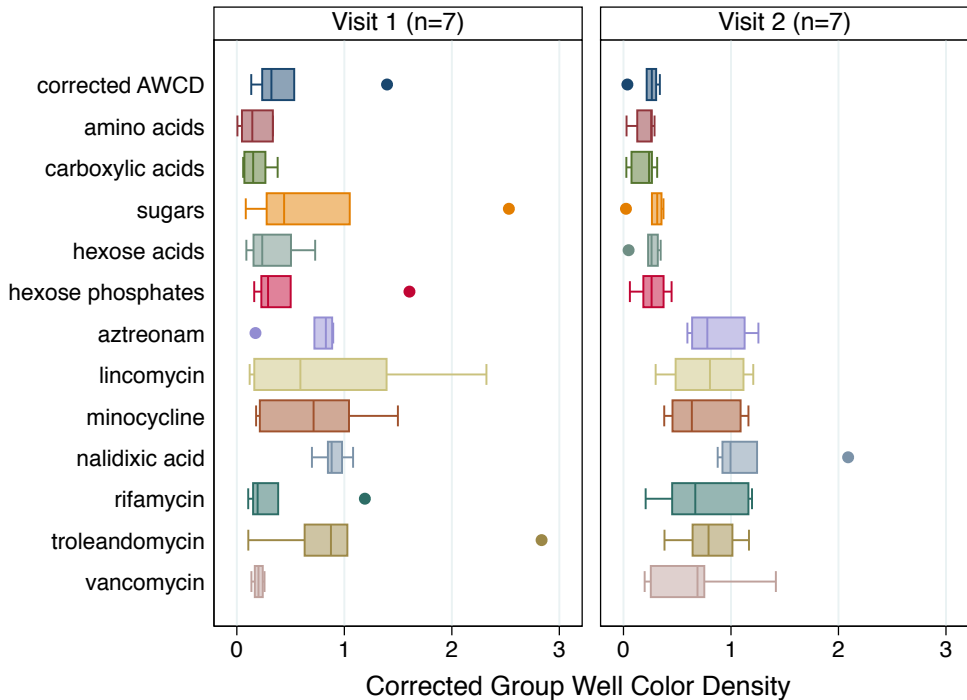

Supplement: Supplementary file 4 — Oxidative activity of sputum samples from seven CF subjects at two clinic visits (paired analysis). Group well color density of metabolic activity corrected for starting bacterial density. P values were generated using Wilcoxon matched-pairs signed-rank tests. No significant differences were noted between clinic visits. (PDF 21 kb) [file 40168_2017_289_MOESM4_ESM.pdf]

Dimension 2

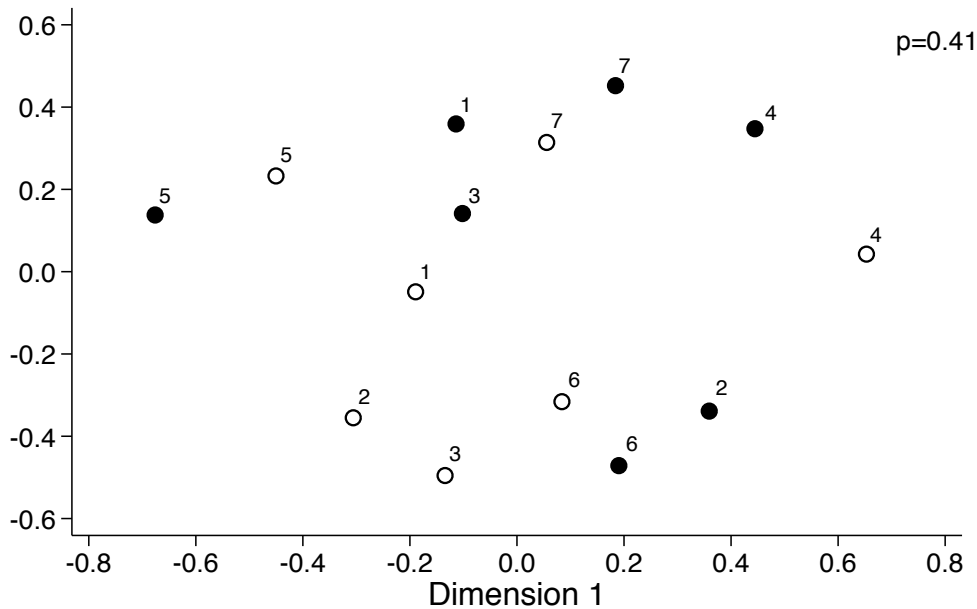

p=0.41

● Visit 1 ○ Visit 2

Supplement: Supplementary file 5 — NMDS ordination plot for seven CF subjects at two clinic visits (paired analysis). Principal coordinate analysis by non-metric multidimensional scaling generated from Bray-Curtis dissimilarity matrices at the genus level from seven CF subjects at two clinic visits. P value was obtained using HOMOVA. No separation was noted between the paired samples. (PDF 139 kb) [file 40168_2017_289_MOESM5_ESM.pdf]

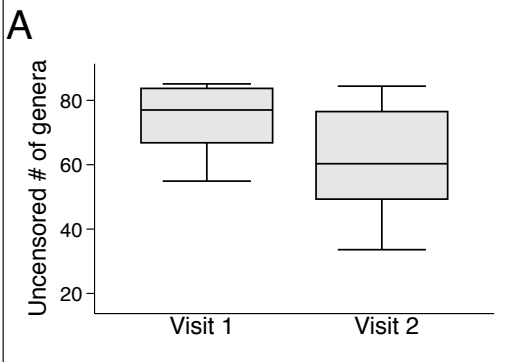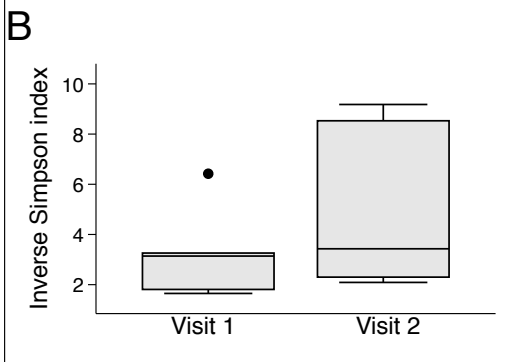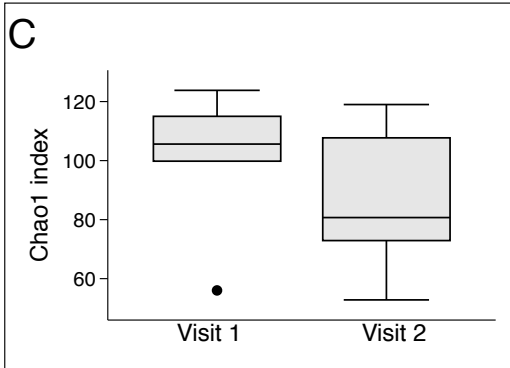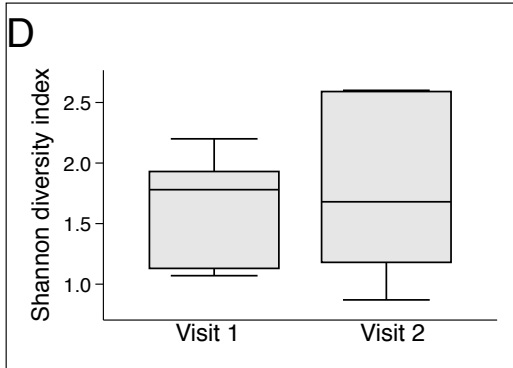

Supplement: Supplementary file 6 — Diversity and richness of CF microbiome in subjects at two clinic visits (paired analysis). Panels show uncensored number of genera, inverse Simpson index, Chao1 index, Shannon diversity Index. P values were generated using Wilcoxon matched-pairs signed-rank tests. No significant differences were noted between clinic visits. (PDF 17 kb) [file 40168_2017_289_MOESM6_ESM.pdf]
